# Cultivation practices and soil water storage effects on grain yield and quality of winter wheat in the Loess Plateau, China

**DOI:** 10.3389/fpls.2025.1735429

**Published:** 2026-01-23

**Authors:** Pengcheng Ding, Hafeez Noor, Xiaofen Li, Jun Xing, Yongqiang Yan, Wendi Guo, Aixia Ren, Linghong Li, Yongkang Ren, Min Sun, Zhiqiang Gao

**Affiliations:** 1 College of Agriculture, Shanxi Agricultural University, Taiyuan, China; 2 Key Laboratory of Sustainable Dryland Agriculture of Shanxi Province, Taiyuan, China; 3 Key Laboratory of Functional Agriculture in the Loess Plateau, Ministry of Agriculture and Rural Affairs, Taigu, Shanxi, China

**Keywords:** dry matter translocation, grain yield, protein, soil water storage, wheat

## Abstract

**Introduction:**

In the water-limited Loess Plateau of China, wheat productivity faces severe constraints. This study investigates the physiological and quality determinants of yield and protein content across multiple winter wheat cultivars to identify key breeding targets for dryland systems.

**Methods:**

Eleven cultivars were analyzed for soil water storage dynamics, dry matter accumulation and translocation, nitrogen use efficiency, and grain quality parameters, including volatile flavor compounds.

**Results:**

High yield potential was driven by superior pre-anthesis nitrogen assimilation and substantial post-anthesis dry matter remobilization. The highest-yielding cultivar (YH-805) achieved this through a greater number of grains per spike. Conversely, higher grain protein content (e.g., in YH-618) was linked to enhanced post-flowering nitrogen translocation. A fundamental yield–protein trade-off was confirmed. The medium-yield, high-protein cultivar YH-115 exhibited the most favorable flavor profile, associated with key volatile compounds like octanal and hexanal.

**Discussion:**

The results demonstrate that yield and quality are governed by distinct pre- and post-anthesis resource allocation strategies. Targeted breeding for specific traits—such as pre-anthesis nitrogen uptake for yield or post-anthesis nitrogen translocation for protein—can help optimize for either enhanced productivity or superior end-use quality in dryland wheat systems.

## 1 Introduction

Winter wheat (*Triticum aestivum* L.) is an important food crop worldwide. It is an important crop in the southeast Loess Plateau of China, accounting for approximately one-fifth of the food production (Noor et al., 2025). The implementation of the reform and opening-up policy, the rapid development of China’s social economy, and the significant improvement in people’s living standards (Noor et al., 2025). In the southeast Loess Plateau and Shanxi Province—major wheat-producing areas—dryland wheat accounts for a large proportion of the cultivated area and contributes substantially to provincial and national production (Kuipers et al., 1994). However, yields in these regions are often unstable because of limited and uneven precipitation during the fallow and growing seasons, variable soil moisture, and constrained agronomic conditions (Noor et al., 2025; Kuipers et al., 1994). Improving yield stability and grain quality under rainfed (dryland) conditions is therefore essential for regional food security and rural livelihoods (Lee et al., 2001). Wheat yield and quality are shaped by the interaction of genotype and environment, together with management practices (Morita et al., 2002). Although advances in breeding and cultivation over recent decades have increased average wheat yields in China, substantial variability remains across varieties and production environments (Apel and Hirt, 2004). The same wheat variety, due to differences in moisture, soil, climate, and cultivation measures, also has different aromas. The reason for this difference is the different types of substances constituting the aroma components and the different contents and proportions of the compounds (Noor et al., 2025; Kuipers et al., 1994; Wang et al., 2020). Therefore, exploring the differences in yield and quality among dryland wheat varieties and optimizing cultivation measures are crucial for improving wheat yield and quality in Shanxi, ensuring food security (Li et al., 2013).

Differences in soil type, moisture availability, climate and management not only influence yield but also affect grain characteristics, such as aroma, protein composition, starch properties and dough functionality—traits that determine processing and end-product quality (e.g., steamed bread and noodles) (Sliwinska et al., 2014). For example, variation in gluten content strongly influences water absorption and dough properties, while starch gelatinization behavior affects cooking and processing quality (Garrido-Delgado et al., 2015). Dryland wheat systems present particular challenges: they account for roughly one-third of China’s cultivated land but produce a disproportionate share of national wheat output, making improvements here especially impactful (Arroyo-Manzanares et al., 2018; Riaz et al., 2018). The goals for modern dryland wheat production have therefore expanded beyond yield to include stability, processing quality, water- and nutrient-use efficiency, and adaptability to marginal conditions (Xi et al., 2021). Screening and promoting high-yielding, high-quality varieties adapted to dryland environments—combined with optimized cultivation practices that improve soil water use, dry-matter accumulation and nutrient uptake—are critical steps toward meeting these goals (Riccardoet al., 2021; Senapati and Semenov, 2020).

This study evaluates yield, water and nitrogen utilization, dry-matter accumulation, and processing quality among key winter wheat varieties grown under dryland conditions in Shanxi. Our objectives are to (1) identify varieties and trait clusters with superior yield and processing quality under rainfed conditions, (2) quantify differences in starch and protein-related quality attributes that affect cooking and dough properties, and (3) propose cultivation measures to improve water use and processing quality in dryland wheat systems. The findings aim to guide variety selection and management practices to enhance both productivity and grain quality in Shanxi’s dryland wheat production.

## 2 Materials and methods

### 2.1 The overview of the experimental site

**Figure 1:**
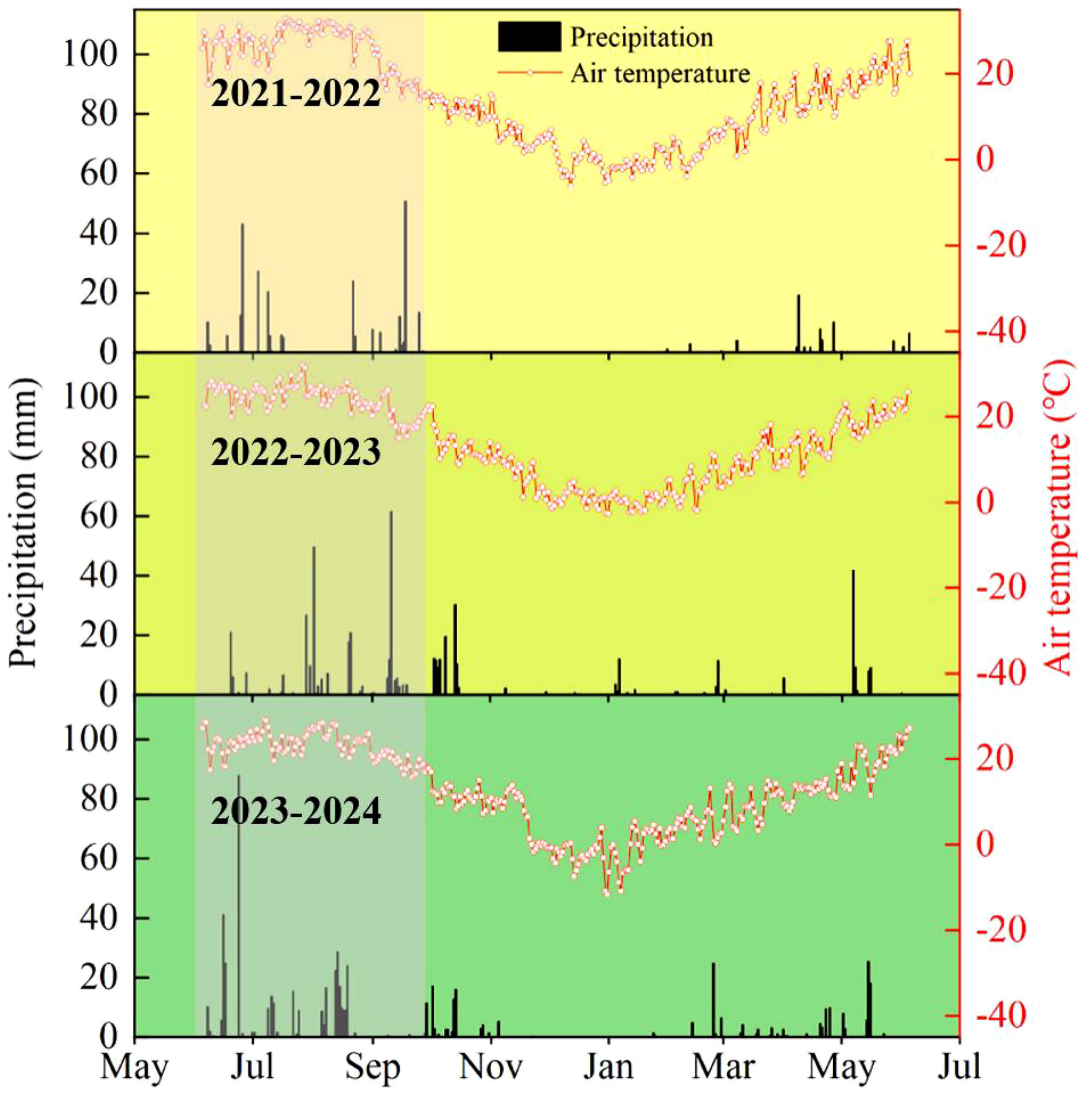
Precipitation and average daily temperature average at different growth stages in the experiment site Wenxi, Shanxi Province 2021–2022, 2022–2023 and 2023–2024 growing seasons. SS, sowing stage; JS, jointing stage; AS, anthesis stage; MS, maturity stage.

The Field experiment was conducted from 2020 to 2022 at the wheat experimental demonstration base in Wenxi, Shanxi Province (35°24′N, 111°26′E), a typical arid area. The average annual temperature is 13.72°C, the average annual sunshine duration is 2461 h. The following sections describe the site characteristics, experimental design, and measurement protocols. Average annual precipitation was normal (**Figure 1**).

### 2.2 Experimental design

The experimental two-factor split-plot design was as follows: Yunhan, 20410; Yunhan, 618; Yunhan, 805; Yunhan, 115; Chang, 6359; Jinmai, 47; Jinmai, 92; Changhan, 58; Changhang, 1; Liangxing, 66; and Luhan, 6. The main information is presented in (**Table 1S**). Each plot measured 300 m2 (6 m × 50 m). Based on the USDA soil textural classification, the study site soils were identified as sandy loam, with a predominance of sand particles, followed by silt, clay fractions. Before sowing, inorganic fertilizers of N fertilizer (urea, 46% N, CNOOC Chemical Co., Ltd., Hainan, China), 150 kg P ha^−1^ as triple super phosphate (16% P_2_O_5_, Yunnan Phosphate Chemical Group Co., Ltd., Kunming, China), and 75 kg K ha^−1^ as potassium chloride (50% K_2_O, Qinghai Salt Lake Industry Co., Ltd., Golmud, China) were spread uniformly and tilled in the top soil layer of 15 cm. The seeding rate was 90 kg ha^−1^. The sowing dates were September 28, 2020, and October 11, 2021, and the harvest dates were June 10, 2021, and June 1, 2022 (**Table 1**). Herbicides and insecticides were applied once in the spring. “One spray for controlling three problems” was also applied at anthesis stage, which was a combination of pesticides, fungicides, plant growth regulators, and micro fertilizer, to prevent diseases, insects, and premature plant aging. Irrigation was not performed during the experiment.

**Table 1:** Soil fertility of 0–20 cm soil layer before sowing in Wenxi field experimental.

Year	Soil depth (cm)	Organic matter (g·kg^–1^)	Available N (mg·kg^–1^)	Available phosphorus (mg·kg^–1^)	Available potassium(mg·kg^–1^)
2020–2021	0-20	13.54	67.39	19.06	96.25
2021–2022	0-20	12.88	69.17	20.89	106.20

### 2.3 Agronomic characteristics

At the jointing, booting, anthesis, and maturity stages, a 0.667 m^2^ plot with uniform growth was randomly selected in each plot to record and investigate the number of wheat plants in the plot, and 10 plants were sampled and divided into two parts. In the anthesis stage, the samples were divided into five parts: leaves, spikes, internodes below the spike, penultimate and other internodes. At the maturity stage, the samples were divided into six parts: leaves, glumes + sheath, grains, internodes below the spike, penultimate internode, and other internodes. The fresh samples were stored in a -20°C refrigerator for subsequent determination of dry samples, killed at deg 105°C for 30 min and then dried at 75°C to a constant weight, weighed, and recorded, except for the grains at maturity stage, which were dried at 65°C to a constant weight dry samples were retained for subsequent determination of plant indicators (Noor et al., 2025).

### 2.4 Grain filling dynamics and post-anthesi dry matter mass

Samples were taken every five days, with 10 plants each time, with three replicates. These were divided into leaves, stems, sheaths, and spikes. The stems were divided into internodes below the spike, penultimate internode, and other internodes according to the internodes. The fresh samples were stored in a –20°C refrigerator. Dry samples were killed at 105°C for 30 minutes and then dried at 75°C to a constant weight, weighed, and recorded (grains were directly dried at 75°C to a constant weight (Noor et al., 2025).

### 2.5 Soil water storage, stage water consumption

Soil drills and samples from the to 0–200 cm of the soil layer were taken at the wheat sowing, jointing, anthesi**s**, and maturity stages. Each 20 cm was considered as one soil layer, and then the samples were placed in a 105°C oven to dry until a constant weight was achieved, and the soil moisture content and soil water storage in different soil layers were calculated. Because the experimental plots were flat, the groundwater depth was greater than 15m and the rainfall infiltration was less than 2 m; thus, the groundwater recharge was ignored. Selecting flat terrain plots, a 2m deep profile was dug, and samples were taken every 20 cm.

### 2.6 Soil textural classification

The soil samples were analyzed for particle size distribution using the [hydrometer method/sieve-pipette method, adjusted according to your actual method]. The results were classified according to the United States Department of Agriculture (USDA) Soil Textural Classification System. This approach allows the standardized categorization of soils into textural classes (e.g., sandy loam, clay loam, silty clay) and provides an internationally recognized framework for comparison across studies.

### 2.7 Nitrogen calculation

Values for nitrogen were calculated following formulae (Tao et al., 2019).


$$\begin{array}{l} \text{Plant nitrogen accumulation}=\text{plant biomass}\times \\ \text{nitrogen content; pre-anthesis accumulated nitrogen }\\ \text{translocation }\left(\text{PANT}\right)=\text{nitrogen}\\ \text{accumulation in vegetative organs at the anthesis stage-nitrogen}\\ \text{ accumulation in vegetative organs at the maturation stage.} \end{array}$$

$$\begin{array}{l} \text{Contribution to N in spikes }\left(\%\right)\text{ of PANT}=\text{PANT}\\ =\text{Nitrogen accumulation in spikes }100\%) \end{array}$$

$$\begin{array}{l} \text{Nitrogen accumulation after anthesis }\left(\text{NAAA}\right)\\ =\text{nitrogen accumulation in the plant at the maturation stage}\\ –\text{nitrogen accumulation in the plant at the anthesis stage} \end{array}$$

$$\begin{array}{l} \text{Contribution to N in spikes }\left(\%\right)\text{ of NAAA}=\text{NAAA}\\ =\text{nitrogen accumulation in kernels }100\% \end{array}$$

$$\begin{array}{c} \text{N uptake efficiency}=\\ \text{nitrogen accumulation in plant}/\text{applied amount of nitrogen} \end{array}$$

N use efficiency=grain yield/nitrogen accumulation in plants

N productive efficiency=grain yield/applied amount of nitrogen

### 2.8 Plant carbohydrates

Dry samples of plants at each growth stage were ground through a 0.15 mm sieve; sucrose content was determined using the resorcinol method, and soluble sugar content of plants was determined using the sulfuric acid-anthrone method (Noor et al., 2025).

### 2.9 Computing method

The total water consumption throughout the entire growth period is calculated as follows:


ET=ΔS+P+G

where ΔS represents the reduction in soil water storage (mm) at each growth stage, P represents effective precipitation (mm), and G represents groundwater recharge (mm). In this experiment, the groundwater depth was below 10 m; therefore, the G value could be ignored.


$$\begin{array}{c} \text{Accumulation of soluble carbohydrates}\\ =\text{Soluble carbohydrate content}\times \text{Dry matter weight} \end{array}$$

$$\begin{array}{c} \text{Dry matter translocation from stem and sheath before flowering}\\ =\text{Dry matter weight of stem and sheath at flowering}\\ -\text{Dry matter weight of stem and sheath at maturity} \end{array}$$

### 2.10 Plant nitrogen

Aboveground plants at anthesis and maturity were sampled from 20 randomly selected plants in each plot for the measurement of stem + sheath, glume + spike, grain, and total plant dry matter. samples were initially oven-dried at 105°C for 30 min and weighed after further drying at 70°C until constant weight was attained. The stem + sheath, glume + spike, grain, and total plant N concentrations of oven-dried, ground, and acid-digested plant samples were determined using the indophenol blue colorimetric method.

### 2.11 Yield and its components

At maturity, 0.667 m2 of wheat with uniform growth was selected for each plot to determine the number of spikes. Twenty spikes were randomly selected from each plot, dried, and the average number of grains per spike was calculated, and the 1000–grain weight was determined with three replicates. 20 m2 were randomly harvested to determine yield. The grain moisture content was determined using a grain moisture meter, and the actual yield was calculated based on the national grain storage standard moisture content of 13% (Tao et al., 2019).

### 2.12 Statistical analyses

Data processing and graphing were performed using Excel 2010, and multiple comparisons (Tukey) of measured parameters were conducted among different cultivars within the same season, as well as among cultivars, with a significance level (α) set at 0.05. Principal component analysis (PCA) was performed using Origin 2018 software (Origin Lab, Northampton, MA, USA), and Pearson’s correlation analysis was conducted using SPSS software to examine the relationships among variables. The least significant difference (LSD) method was used, and the significance level was set at =0.05.

## 3 Results

### 3.1 Characteristics of plant dry matter translocation

**Figure 2:**
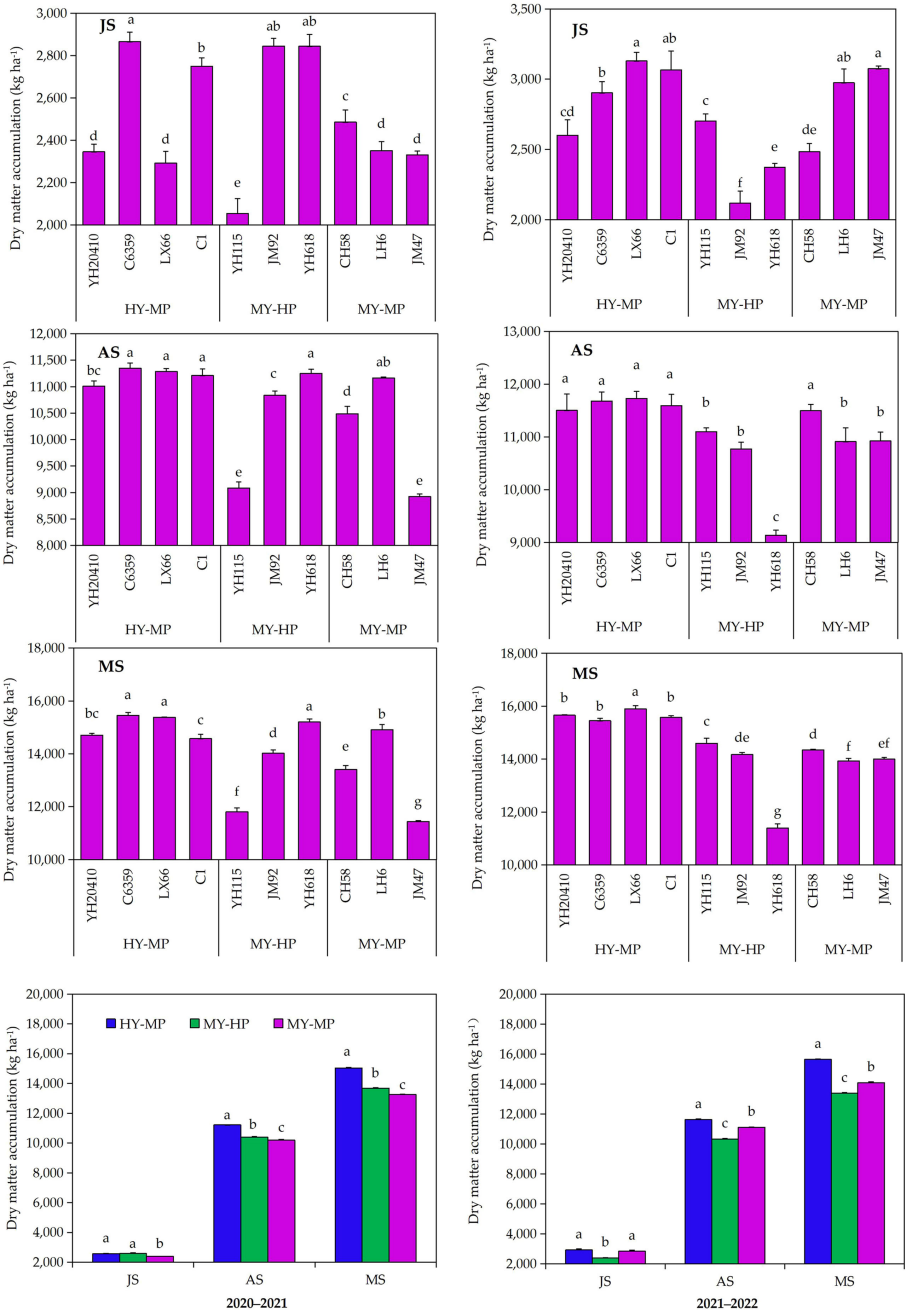
Difference of dry matter translocation at three growth stages in different wheat cultivars. Jointing stage, JS; anthesis stage, AS; mature stage, MS. HY-MP means that wheat varieties with high yield and medium protein content, MY-HP means that wheat varieties with yield medium and high protein content, MY-MP means that wheat varieties with medium yield and medium protein content.

The high–yield, protein-rich varieties compared with medium-yield protein-rich varieties, medium-yield protein varieties had lower dry matter translocation during the jointing, anthesis, and maturity stages (**Figure 2**) 2020–2021. Among them, the dry matter translocation at the jointing stage was the highest in the high-yield protein variety LX–66, and there were significant differences compared with the other treatments, except for the high–yield protein variety C–1, the medium-yield protein variety LH–6, and JM–47. The dry matter translocation at the anthesis stage was the highest in the high-yield protein variety LX–66, and there were significant differences compared with the other treatments except for the high-yield protein variety CH–1, YH–20410, C–6359. In The medium–yield protein variety YH–115 and the medium-yield protein and medium–yield protein varieties LH–6 and CH–58, the dry matter accumulation at the maturity stage was the highest in the high-yield protein variety LX–66, and there were significant differences compared with other treatments. The dry matter content of the medium-yield protein varieties was lower than that of the high-yield protein varieties during the jointing, anthesis, and maturity stages.

### 3.2 Soil water storage in the 0–200 cm layer at each growth stage

**Figure 3:**
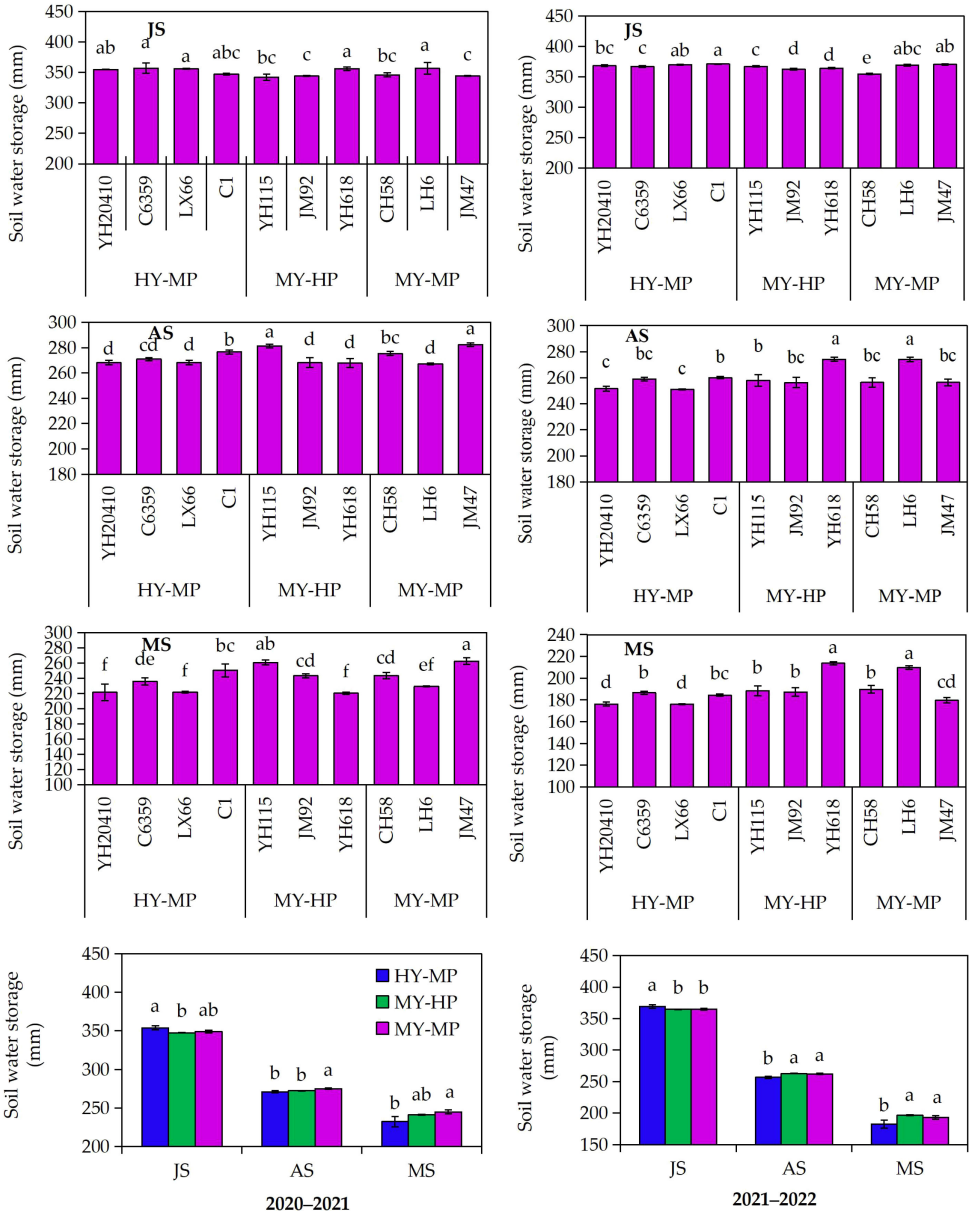
The difference of soil water storage of 0–200 cm in different cultivars of dryland wheat. Jointing stage, JS; anthesis stage, AS; mature stage, MS.

The medium-yield high-protein variety had higher soil water storage at anthesis and maturity than the high-yield medium-protein variety, with no significant difference at anthesis but a decrease at jointing 2020–2021 (**Figure 3**). At jointing, medium–yield medium-protein C–6359 had the highest soil water storage, which was significantly different from the other three high–yield medium-protein varieties, YH–618 and LH–6. At anthesis and maturity, the medium-yield medium-protein variety JM–47 had the highest soil water storage, which was significantly different from all treatments, except the medium-yield high-protein variety YH–618. In 2021–2022, the medium–yield high-protein variety had higher soil water storage at anthesis and maturity than the high-yield medium-protein variety, with no significant difference at anthesis. At jointing, the medium-yield medium-protein variety YH–115 had the highest soil water storage, which was significantly different from the high-yield medium-protein variety LX–66 and the other medium-yield medium-protein variety LH–6. Anthesis and maturity, the medium-yield high-protein variety YH–618 had the highest soil water storage, which was significantly different from all treatments except the medium-yield medium-protein variety YH–115 and LH–6, and was also significantly different from all treatments except the medium–yield high-protein variety YH–115. The medium-yield, high-protein variety YH–115, along with other varieties in its category, generally have higher soil water storage at anthesis and maturity. In contrast, high-yield, medium-protein varieties had higher water storage at the jointing stage.

### 3.3 Varietal and annual effects on dry matter dynamics

Wheat varieties exhibited a highly significant effect on key dry matter parameters: the amount of pre-anthesis dry matter translocated (Pre-T), the accumulation of post-anthesis dry matter (Post-A), and the contribution rate of each process to final grain weight (**Table 2**). Furthermore, a significant year × variety interaction was observed for the contribution of pre-anthesis translocation, as well as for both the accumulation and contribution of post-anthesis dry matter. Across varieties and years, the grain dry matter was derived from two sources: translocation of assimilates stored before anthesis and accumulation of new assimilates after anthesis. Pre-anthesis translocation contributed 27.76% to 44.39% of total grain dry matter. In contrast, post-anthesis accumulation, ranging from 2009.41 to 4132.83 kg ha^-^¹, was the predominant source, contributing 55.61% to 72.24%. Significant differences existed among varieties for all measured dry matter parameters. Notably, post-anthesis dry matter accumulation was the primary determinant of final grain yield. In the 2022–2023 season, its Post-A (4132.83 kg ha^-^¹) was significantly higher than all other varieties. In the 2023–2024 season, it again recorded the highest Post-A (4023.38 kg ha^-^¹), showing no significant difference only with YH–20410, C–1, LX–66, and JM–47, but remaining significantly higher than the rest.

**Table 2:** Differences of dry matter translocation before anthesis and dry matter translocation after anthesis under different cultivars on dryland winter wheat.

Year	Cultivars	DMABA		DMAAA	
		TA (kg·ha^–1^)	CG (%)	AA (kg ha ^–1^)	CG (%)
2020–2021	YH–618	1715.27 bc	31.25 de	3772.79 bc	68.75 ab
	YH–805	1587.99 cd	27.76 f	4132.83 a	72.24 a
	YH–20410	1777.78 b	33.73 cd	3492.18 de	66.27 bc
	LH–6	1650.63 bc	31.22 de	3635.63 cd	68.78 ab
	JM–92	1705.58 bc	36.35 bc	2986.83 f	63.65 cd
	C–6359	1740.97 b	34.14 cd	3358.54 e	65.86 bc
	CH–1	1751.11 b	38.47 b	2801.26 g	61.53 d
	CH–58	1959.48 a	42.21 a	2682.89 g	57.79 e
	LX–66	1633.11 bc	29.7 ef	3865.58 b	70.3 a
	JM–47	1391.49 e	36.42 bc	2429.71 h	63.58 cd
	YH–115	1451.12 de	38.47 b	2321.28 h	61.53 d
	Mean	1669.50	34.52	3225.41	65.48
2021–2022	YH–618	1579.11 c	44.00 a	2009.41 d	56.00 c
	YH–805	1593.94 c	28.38 d	4023.38 a	71.62 a
	YH–20410	1631.35 c	29.44 cd	3910.33 a	70.56 a
	LH–6	1974.10 ab	44.39 a	2473.45 c	55.61 c
	JM–92	1631.95 c	34.11 bc	3152.64 b	65.89 a
	C–6359	1806.44 abc	35.91 b	3224.41 b	64.09 ab
	CH–1	1720.01 bc	31.52 bcd	3737.25 a	68.48 a
	CH–58	2029.19 a	43.81 a	2602.4 c	56.19 bc
	LX–66	1662.48 c	29.77 cd	3922.34 a	70.23 a
	JM–47	1578.28 c	29.03 cd	3859.35 a	70.97 a
	YH–115	1585.97 c	32.78 bcd	3252.00 b	67.22 a
	Mean	1708.44	34.83	3287.90	65.17
ANOVA					
Year (Y)		ns	ns	ns	ns
Cultivars (C)		**	**	**	*
Y × C		ns	**	**	**

Dry matter translocation before anthesis (DMABA) and dry matter translocation after anthesis (DMAAA), Total assimilates allocated (TAA), Contribution to grain yield (CG). Different lowercase letters represent significant differences (*p ≤* 0.05). Different letters indicate that means within a column are significantly different.

**, * significant at p < 0.01 and p < 0.05, respectively; ns, not significant. Means within columns followed by different lower case letters are significantly different (p < 0.05, LSD test).

### 3.4 Nitrogen metabolism before after anthesis and nitrogen accumulation after anthesis and their contributions to grains

The year and variety had significant or extremely significant effects on nitrogen transport volume before anthesis, N accumulation after anthesis, and contribution rate to grains (**Table 3**). The N transported from the vegetative organs to the grains before anthesis was 62.6 kg ha^–1^ - 125.68 kg ha^–1^, accounting for 65.19%-85.21% of the nitrogen accumulation in grains. The N accumulation after anthesis was 19.22 kg ha^–1^, 38.28 kg ha^–1^, accounting for 15.52%, 37.41% of the N accumulation in grains. There were differences in the N accumulation volume before anthesis, nitrogen transfer volume to grains after anthesis, and contribution rate of N to grains among drought-tolerant wheat varieties. In 2020 to 2021, the N transport volume after anthesis was significantly the highest in YH–618, reaching 38.28 kg ha^–1^. In 2021 to 2022, the N transport volume after anthesis was significantly the highest in YH–618, reaching 34.88 kg ha^–1^. The average nitrogen transport volume before anthesis was the highest in YH–805, reaching 125.64 kg ha^–1^. The higher N transport volume after anthesis of YH–618 was the main reason for its higher protein content in grains, while the higher N transfer volume before anthesis and the contribution rate to grains were the main reasons for the high yield of YH–805.

**Table 3:** Differences of N translocation before anthesis and N accumulation after anthesis in different cultivar wheat.

Year	Cultivars	PANT		NAAA	
		Amount of translocation (kg ha^-1^)	Contribution to N in grains (%)	Amount of accumulation (kg ha^-1^)	Contribution to N in grains (%)
2020–2021	YH–618	113.87 b	74.84 b	38.28 a	24.13 c
	YH–805	124.40 a	81.88 a	27.54 c	21.10 d
	YH–20410	100.31 c	79.38 a	26.05 cd	23.44 d
	LH–6	100.55 c	80.51 a	24.34 cd	20.72 d
	JM–92	82.67 d	70.27 c	34.98 ab	27.55 b
	C–6359	102.19 c	78.22 ab	28.46 c	17.57 cd
	CH–1	76.74 e	67.24 cd	37.39 ab	32.91 ab
	CH–58	78.65 de	68.25 cd	36.59 ab	30.44 ab
	LX–66	99.12 c	81.48 a	22.52 d	19.72 d
	JM–47	67.40 f	65.81 d	35.02 ab	35.34 a
	YH–115	62.60 g	65.19 d	33.43 b	37.41 a
	Mean	91.68	73.91	31.33	26.09
2021–2022	YH–618	70.50 h	66.90 f	34.88 a	30.11 a
	YH–805	125.68 a	83.27 ab	25.26 cde	15.52 ef
	YH–20410	110.74 b	85.21 a	19.22 f	12.54 f
	LH–6	70.59 h	71.15 e	28.63 bc	27.58 b
	JM–92	98.89 de	76.86 cd	29.78 b	22.91 c
	C–6359	94.55 ef	75.83 d	30.14 b	25.76 c
	CH–1	111.83 b	82.42 ab	23.85 e	18.30 def
	CH–58	85.93 g	75.14 d	28.43 bcd	22.82 c
	LX–66	103.63 c	80.54 bc	25.03 de	21.22 de
	JM–47	102.19 cd	80.11 bc	25.38c de	17.48 d
	YH–115	90.71 f	70.62 ef	37.73 a	30.16 b
	Mean	96.84	77.05	28.05	22.95
ANOVA					
Year (Y)		ns	ns	*	*
Cultivars (C)		**	**	**	ns
Y × C		*	**	**	**

PANT, pre-anthesis accumulated nitrogen translocation (amount moved from vegetative organs to kernels after anthesis); NAAA, nitrogen accumulation after anthesis. Different lowercase letters represent significant differences (*p ≤* 0.05). Different letters indicate that means within a column are significantly different.

**, * significant at p < 0.01 and p < 0.05, respectively; ns, not significant. Means within columns followed by different lower case letters are significantly different (p < 0.05, LSD test).

### 3.5 Nitrogen accumulation in various organs of during anthesis and maturity periods and nitrogen use efficiency

The year had significant or extremely significant effects on the N accumulation in leaves during anthesis, stem + sheath + glume, leaves during maturity, stem + sheath + glume, and grains (**Table 4**). The interaction between variety and year also had significant or extremely significant effects on the N accumulation in various organs during anthesis and maturity. In 2020-2021, the N accumulation amount of each organ during anthesis was the highest in YH–805, with 30.79 kg ha^–1^ for leaves, 100.17 kg ha^–1^ for stems + sheaths, and 25.81 kg ha^–1^ for sheath + stem. There were significant differences among the varieties, except for YH–805, Chang6359 and LX–66, there were significant differences. The nitrogen accumulation amount during anthesis of YH–805 was the highest, reaching 156.77 kg ha^–1^, and there were significant differences with the other varieties. Nitrogen accumulation during anthesis was the highest in YH–805, reaching 154.32 kg ha^–1^, and there were significant differences with other varieties. The nitrogen accumulation amount of grains was significantly the highest in YH–618, reaching 152.15 kg ha^–1^, and there were significant differences among all varieties, except YH–805. In 2021-2022, the nitrogen accumulation amount of grains was significantly the highest in YH–805, reaching 179.57 kg ha^–1^, and there were significant differences with the other varieties. The average nitrogen accumulation amount of grains in the two years was significantly the highest in YH–805, reaching 150.93 kg ha^–1^, and there were significant differences with the other varieties. The N accumulation in various organs of YH–805 during flowering was conducive to promoting the transfer of nitrogen from leaves and sheath + stem + glume to grains, and YH–805 and YH–618 had higher amounts of accumulated grain and nitrogen during maturity.

**Table 4:** Differences of nitrogen accumulation in various organs of wheat at the anthesis and maturation stages of different cultivar wheat.

Year	Cultivars	Anthesis				Maturity		
		Leaf	Stem+sheath	Glume+spike	Leaf	Stem+sheath	Glume+spike	Grain
2020–2021	YH–618	28.18 b	92.63 b	25.09 ab	5.78 cd	16.97 ab	9.28 a	152.15 b
	YH–805	30.79 a	100.17 a	25.81	6.23 c	17.26 a	8.87 b	151.94 a
	YH–20410	24.34 c	81.82 c	22.15 cd	5.19 e	16.84 ab	5.97 f	126.36 cd
	LH–6	24.80 c	82.82 c	23.51 bc	7.42 b	16.12 bc	7.04 c	124.89 d
	JM–92	19.93 de	68.38 d	21.18 de	5.25 e	15.09 cd	6.47 de	117.66 ef
	C–6359	25.12 c	82.28 c	24.16 ab	8.20 a	14.30 de	6.85 cd	130.65 c
	CH–1	19.91 de	63.60 e	18.28 f	5.22 e	13.08 f	6.75 cd	114.13 f
	CH–58	20.80 d	64.29 e	20.33 e	5.77 d	14.86 d	6.14 ef	115.23 f
	LX–66	25.32 c	81.80 c	24.02 ab	8.46 a	16.50 ab	7.05 c	121.64 de
	JM–47	18.46 ef	57.99 f	16.97 fg	6.51 c	13.37 ef	6.14 ef	102.42 g
	YH–115	17.50 f	55.08 g	16.02 g	5.92 d	11.84 g	8.24 b	96.02 h
	Mean	23.2	75.53	21.59	6.36	15.11	7.17	123.01
2021–2022	YH–618	16.81 g	56.56 g	17.03 c	2.03 fg	12.07 c	5.80 h	105.37 f
	YH–805	30.07 a	98.21 a	26.04 a	4.81 b	15.94 a	7.89 cd	150.93 a
	YH–20410	27.20 b	86.09 bc	24.11 ab	4.37 c	15.73 a	6.56 g	129.96 c
	LH–6	17.47 g	60.21 g	17.82 c	2.36 ef	15.19 ab	7.37 ef	99.21 g
	JM–92	21.23 ef	77.66 de	24.48 ab	2.59 e	14.86 ab	7.03 fg	128.67 c
	C–6359	20.29 f	74.6e	24.83 ab	3.57 d	12.61 c	9.00 a	124.68 d
	CH–1	25.40 bc	87.06 b	25.79 a	1.72 g	15.93 a	8.78 a	135.68 b
	CH–58	20.36 f	69.50 f	22.3 b	3.25 d	14.88 ab	8.11 bc	114.36 e
	LX–66	23.73 cd	81.81 cd	24.19 ab	5.59 a	12.88 c	7.63 cde	128.67 c
	JM–47	22.82 de	81.04 d	25.39 a	4.67 bc	14.81 ab	7.59 de	127.56 cd
	YH–115	21.53 ef	73.58 ef	22.47 b	4.58 bc	13.70 bc	8.59 ab	128.44 c
	Mean	22.45	76.94	23.13	3.59	14.42	7.67	124.89
ANOVA								
Year (Y)		*	ns	*	**	ns	**	*
Cultivars (C)		**	**	**	**	**	**	**
Y × C		**	**	**	**	**	**	ns

Different lowercase letters represent significant differences (*p ≤* 0.05). Different letters indicate that means within a column are significantly different.

**, * significant at p < 0.01 and p < 0.05, respectively; ns, not significant. Means within columns followed by different lower case letters are significantly different (p < 0.05, LSD test).

The year had a highly significant effect on N absorption efficiency, whereas the variety had a highly significant effect on N absorption efficiency, N harvest index, nitrogen utilization efficiency, and N production efficiency (**Table 5**). The interaction between year and variety also had a highly significant effect on N absorption efficiency, N utilization efficiency, and N production efficiency. In the 2020–2021 growing season, the N absorption efficiency of dryland wheat was significantly higher for YH–618 than for all other varieties except YH–805. The N harvest index was also the highest for YH–618, reaching 0.83, which was significantly higher than LX–66 the N production efficiency of YH–618 was significantly, and was significantly higher than that of the other treatments. In the 2021–2022 N absorption efficiency was significantly highest for YH–805, reaching was significantly higher than that of other varieties the N harvest index was also the N production efficiency of YH–618 was significantly the highest.

**Table 5:** Differences of nitrogen use efficiency traits of different cultivar wheat.

Year	Cultivars	N absorption efficiency (kg ha^–1^)	N Harvest Index	N use efficiency (kg ha^–1^)	N productive efficiency
2020–2021	YH–618	1.23 a	0.83 a	29.80 e	36.59 b
	YH–805	1.23 a	0.82 a	31.04 d	38.14 a
	YH–20410	1.03 c	0.82 ab	34.14 b	35.13 c
	LH–6	1.04 bc	0.80 ab	34.00 b	35.24 c
	JM–92	0.96 d	0.81 ab	32.48 c	31.28 e
	C–6359	1.07 b	0.82 ab	31.87 cd	34.00 d
	CH–1	0.93 e	0.82 ab	32.71 c	30.35 e
	CH–58	0.95 de	0.81 ab	32.69 c	30.95 e
	LX–66	1.02 c	0.79 b	35.79 a	36.66 b
	JM–47	0.86 f	0.80 ab	29.75 e	25.47 f
	YH–115	0.81 g	0.79 b	30.92 d	25.15 f
	Mean	1.01	0.81	32.29	32.63
2021–2022	YH–618	0.84 f	0.84 a	28.64 e	23.92 e
	YH–805	1.20 a	0.84 ab	31.28 d	37.45 a
	YH–20410	1.04 c	0.83 abc	35.38 ab	36.94 a
	LH–6	0.83 f	0.80 d	35.83 a	29.65 d
	JM–92	1.02 cd	0.84 ab	31.24 d	31.90 bc
	C–6359	1.00 d	0.83 abc	33.57 bc	33.54 b
	CH–1	1.08 b	0.84 ab	33.66 bc	36.38 a
	CH–58	0.94 e	0.81 cd	32.94 cd	30.88 cd
	LX–66	1.03 c	0.83 abc	36.09 a	37.23 a
	JM–47	1.03 c	0.82 bc	35.17 ab	36.25 a
	YH–115	1.04 c	0.83 abc	31.15 d	32.25 bc
	Mean	1.00	0.83	33.18	33.31
ANOVA					
Year (Y)		*	**	ns	ns
Cultivars (C)		**	*	*	**
Y × C		**	ns	**	**

Different lowercase letters represent significant differences (*p ≤* 0.05). Different letters indicate that means within a column are significantly different.

**, * significant at p < 0.01 and p < 0.05, respectively; ns, not significant. Means within columns followed by different lower case letters are significantly different (p < 0.05, LSD test).

### 3.6 The yield of dryland wheat and the differences among its varieties

The year has a highly significant effect on the number of ears and the 1000-grain weigh, while the variety has a highly significant effect on the yield, the number of ears, the number of grains per ear, and the 1000-grain weigh the interaction between the year and the variety has a highly significant effect on the yield, the number of ears, the number of grains per ear, and the 1000-grain weighs (**Table 6**). In 2020-2021, the yield of dryland wheat was significantly highest for YH–805, reaching 5720.82 kg ha^–1^, which was significantly different from that of the other varieties. The yield of dryland wheat was also highest for YH–805, reaching 5617.32 kg ha^–1^, and it was significantly different from all varieties except YH–20410, C–1, LX–66, and JM–47. The average yield of the two years was highest for YH–805, reaching 5669.07 kg ha^–1^. The grain yield of YH–805 was higher than that of the other wheat varieties. The average number of ears per unit area was highest for LX–66, reaching 666.3×104 ha^–1^, which was significantly different from the other varieties (**Table 7**). The number of grains per ear was the highest for JM–92 in 2021-2022, reaching 35.3, and it was significantly different from YH–618, YH–805, LH–6, and C–6359. The number of grains per ear was also the highest for YH–805, reaching 35.2, and was significantly different from C–6359 and C–1. The average number of grains per ear over the two years was the highest for L–6, reaching 35.0 g, which was significantly higher than that of the other treatments. YH–805 was not significantly different in yield from the varieties YH–20410 and C–6359. However, its main advantage over other varieties is the higher number of grains per ear, which is the primary reason for its higher yield.

**Table 6:** Grain yield and components of different cultivar on dryland winter wheat.

Year	Cultivars	Grain yield (kg ha^–1^)	Spike number (10^4^ ha^–1^)	Grain number per spike	1000–grain weight (g)
2020–2021	YH–618	5488.06 b	559.00 b	32.50 ab	34.78 ef
	YH–805	5720.82 a	550.00 b	34.87 a	38.99 cd
	YH–20410	5269.97 c	464.00 cd	28.83 bcd	41.23 b
	LH–6	5286.26 c	490.50 c	34.90 a	43.60 a
	JM–92	4692.41 e	358.00 e	35.30 a	38.53 cd
	C–6359	5099.51 d	448.00 cd	31.42 abc	40.15 bc
	CH–1	4552.37 e	453.50 cd	29.98 bcd	39.48 bcd
	CH–58	4642.37 e	418.00 d	27.87 cd	37.58 d
	LX–66	5498.69 b	690.50 a	22.35 e	32.53 f
	JM–47	3821.2 f	452.00 cd	30.6 bcd	34.91 e
	YH–115	3772.4 f	484.00 c	27.08 d	34.82 e
	Mean	4894.91	488.05	30.52	37.87
2021–2022	YH–618	3588.52 e	356.00 de	25.77 e	38.62 g
	YH–805	5617.32 a	419.00 cd	35.17 a	42.39 ef
	YH–20410	5541.67 a	458.00 bc	30.05 bc	48.29 ab
	LH–6	4447.55 d	285.00 f	26.80 de	49.30 a
	JM–92	4784.59 bc	521.00 b	26.75 de	41.33 ef
	C–6359	5030.84 b	307.50 ef	32.52 ab	49.10 a
	CH–1	5457.26 a	417.00 cd	33.50 a	45.40 cd
	CH–58	4631.59 cd	419.00 cd	24.87 e	45.09 cd
	LX–66	5584.81 a	642.00 a	24.63 e	46.37 bc
	JM–47	5437.63 a	454.00 c	28.90 cd	43.56 de
	YH–115	4837.97 bc	423.50 c	29.22 cd	41.05 fg
	Mean	4996.34	419.32	28.92	44.59
ANOVA					
Year (Y)		ns	**	ns	*
Cultivars (C)		**	**	**	**
Y × C		**	**	**	**

**, * significant at p < 0.01 and p < 0.05, respectively; ns, not significant. Means within columns followed by different lower case letters are significantly different (p < 0.05, LSD test).

**Table 7:** Significance analysis of interactive effects of year planting patterns on the yield components, and water use efficiency.

Parameter	Spike number (10^4^ ha^–1^)	Grain per spike	1000–grain weighs (g)	Grain yield (kg ha^–1^)
Year (Y)	590.967**	266.230**	944.841**	69.342*
Cultivars (C)	26.682**	11.301**	4.094	27.107**
Y×C	9.216**	2.001	45.474**	6.851**
C×Y	4.475**	3.415**	30.417**	52.885**

Different letters indicate that means within a column are significantly different * and ** indicate significance at *p* < 0.05 and *p* < 0.01, respectively.

### 3.7 Evaluation of bread texture

To investigate the differences in flavor quality among these wheat varieties, we invited 15 evaluators to conduct a comprehensive evaluation of bread made from 10 wheat varieties according to the requirements of the experimental sensory indicators. The comprehensive scoring results are shown in (**Table 8**). From the statistical analysis, it can be clearly seen that the average score of YH–115 was the highest at 86.16, whereas the average score of YH–20410 was the lowest at 80.47. Therefore, through the evaluation of flavor and texture, we found that YH–115 had the best flavor and texture, while YH–20410 had the worst flavor and texture.

**Table 8:** Scoring method for sensory evaluation for different cultivars on dryland wheat steamed bread.

Cultivars	Appearance	Surface structure	Appearance and shape	Inner structure	Resilience	Elasticity	Stickiness	Aroma /flavor	Total
YH–618	13.14	11.86	11.60	13.71	7.65	8.89	9.00	8.29	84.14
YH–20410	13.57	9.69	10.14	13.00	8.72	8.74	8.61	8.00	80.47
LH–6	13.29	9.86	11.59	13.86	8.65	8.61	9.00	8.14	83.00
JM–92	12.86	11.86	11.71	13.86	8.30	8.26	8.43	7.86	83.14
C–6359	12.57	10.29	10.29	14.00	8.65	8.84	8.29	8.43	81.36
CH–1	12.00	10.57	12.00	14.14	8.32	8.12	8.14	8.57	81.86
CH–58	12.71	9.43	11.86	13.71	8.53	8.33	8.57	7.43	80.57
LX–66	12.86	11.14	12.43	13.00	8.20	8.50	8.29	8.29	82.71
JM–47	12.29	12.00	11.71	13.29	7.56	7.86	8.29	8.43	81.43
YH–115	12.71	13.43	12.86	13.57	8.47	8.25	8.71	8.86	86.86

### 3.8 Starch accumulation in grains

There are differences in amylose, amylopectin, total starch content, and the straight/branched ratio in the grains of different wheat varieties. In 2020-2021, the contents of amylose, amylopectin, and total starch in CH–1 were the highest, followed by C–6359, CH–58, and YH–805. In 2021-2022, JM–92 had the highest amylose, amylopectin, and total starch content, which was significantly different from other varieties, except Yunhan618. The two-year average amylose, amylopectin, and total starch contents in grain were the highest in JM–92 and the lowest in CH–58 (**Table 9**). In 2020-2021, the grain starch yield of LX–66 was the highest, which was significantly different from that of the other varieties. In 2021-2022, Yunhan 20410 had the highest starch yield, which was significantly different from that of the other varieties. The two-year average grain starch yield of Liangxing 66 was the highest (3484.85 kg ha^-1^), followed by Yunhan20410 (3325.84 kg ha^-1^).

**Table 9:** Differences of starch content of different cultivar of dryland winter wheat.

Year	Cultivars	Am (%)	Ap (%)	Starch (%)	Am/Ap	Starch yield (kg ha^–1^)
2020-2021	YH–618	13.25 cd	46.40 de	59.65 d	0.29 a	3273.68 bc
	YH–805	12.21 f	44.57 f	56.79 f	0.27 bc	3248.63 c
	YH–20410	12.60 e	46.00 e	58.60 e	0.27 bc	3088.20 e
	LH–6	12.89 de	46.73 d	59.62 d	0.28 bc	3151.78 d
	JM–92	13.56 bc	47.47 c	61.03 c	0.29 a	2863.78 g
	C–6359	14.19 a	50.41 a	64.60 a	0.28 ab	3294.22 b
	CH–1	14.22 a	50.83 a	65.05 a	0.28 ab	2961.20 f
	CH–58	11.82 g	42.72 g	54.54 g	0.28 abc	2532.16 h
	LX–66	13.70 b	49.48 b	63.18 b	0.28 abc	3473.97 a
	JM–47	13.37 bc	49.46 b	62.83 b	0.27 c	2400.78 i
	YH–115	12.70 e	46.54 de	59.24 d	0.27 bc	2234.69 j
	Mean	13.14	47.33	60.47	0.28	2956.64
2021-2022	YH–618	13.82 ab	51.22 ab	64.94 ab	0.27 a	2330.5 i
	YH–805	12.63 d	47.28 f	59.71 e	0.27 a	3353.92 c
	YH–20410	13.49 dc	51.19 ab	64.60 b	0.26 ab	3580.11 a
	LH–6	13.21 c	50.85 bc	64.12 b	0.26 bc	2851.78 g
	JM–92	13.90 a	51.99 a	65.78 a	0.27 a	3147.50 d
	C–6359	12.51de	48.78 de	61.22 d	0.26 c	3079.96 e
	CH–1	12.61d	48.53 e	61.07 d	0.26 bc	3332.56 c
	CH–58	12.07 f	45.02 gh	57.15 f	0.27 a	2646.89 h
	LX–66	12.69 d	49.88 cd	62.69 c	0.25 c	3501.33 b
	JM–47	11.52 g	44.30 h	55.72 g	0.26 bc	3029.60 f
	YH–115	12.26 ef	46.09 g	58.3 f	0.27 ab	2820.72 g
	Mean	12.79	48.65	61.39	0.26	3061.35
ANOVA						
Y		56.363 *	204.152 **	69.159 *	131.489 **	599.314 **
C		45.417 **	88.763 **	142.113 **	4.053 **	1277.192 **
Y × C		37.095 **	67.203 **	109.404 **	2.688 *	674.561 **

Different letters indicate that means within a column are significantly different (*p ≤ 0.05*), * and ** indicate signiffcance at *P ≤ 0.05* and *P ≤ 0.01*, respectively.

### 3.9 The relative content of volatile substances in wheat flour

**Figure 4:**
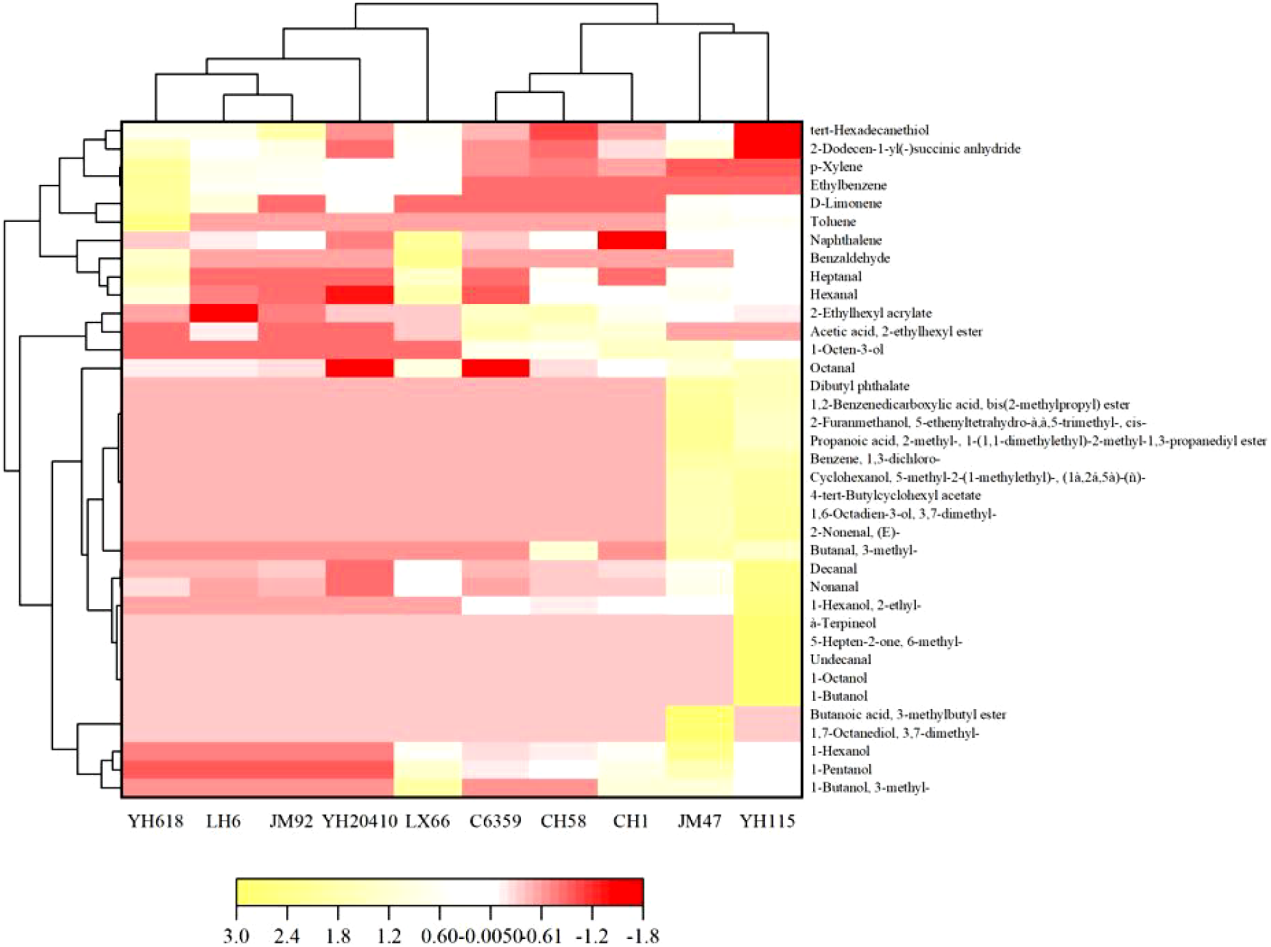
Systematic cluster analysis diagram of volatile compounds in different cultivars on dryland wheat.

The volatile substances in 10 types of wheat flour, namely YH–20410, YH–618, JM–92, LH–6, C–6359, CH–1, CH–58, LX–66, JM–47, and YH–115, were determined using headspace solid-phase microextraction combined with gas chromatography and mass spectrometry (**Table 10**). A total of 74 volatile flavor substances were identified and classified, including eight aliphatic substances: n-butanol, 3-methyl-1-butanol, n-pentanol, n-hexanol, 1-octen-3-ol, 2-ethylhexanol, linalool alcohol, and n-octanol. Nine aldehyde substances, namely 3-methylbutanal, n-hexanal, heptanal, benzaldehyde, octanal, nonal, cis-2-nonen-1-ol, decanal, and undecanal; one ketone substance, namely 6-methyl-5-hepten-2-one 7 ester substances, namely isopentyl butyrate, 2-ethylhexyl acetate, acrylic acid-2-ethylhexyl ester, 4-tert-butylcyclohexyl acetate, 2,4,4-trimethylpentane-1,3-diyldi (2-methylpropanoate), isophthalic acid diisobutyl ester, isophthalic acid dibutyl ester 5 terpene substances, namely D-limonene, (S)-oxygermacrolide, geraniol, DL menthol, and α-pinene 5 benzene derivatives, namely toluene, ethylbenzene, ortho-xylene, 1,3-dichlorobenzene, and naphthalene (**Figure 4**); and the other two types, namely succinic anhydride and octadecane (37 types). Analysis of the composition ratios and components of these substances showed that aldehyde, alcohol, and alkane substances constitute the main volatile component groups in wheat flour. We found that no alcohols were detected in the wheat flour of YH–20410, and the types and relative contents of aldehyde and terpene substances were the lowest, while the types and relative contents of alkanes were higher. In the wheat flour of YH–115, the types and relative contents of alcohols, aldehydes, ketones, terpenes, and esters were higher, while the types and relative contents of alkanes were the lowest.

**Table 10:** The flavor analysis results of different cultivars on dryland wheat flour.

Cultivars	Floure	Alcohols	Aldehydes	Ketones	Benzene derivatives	Terpenes	Esters	Others	Alkanes	Total
YH–618	Relative amount RC (%)	—	14.01	—	7.67	0.78	1.52	0.82	75.20	100
	Kind No.	—	6	—	4	1	1	2	27	41
YH–20410	Relative amount RC (%)	—	3.05	—	2.58	0.35	1.75	0.51	91.76	100
	Kind No.	—	2	—	3	1	1	2	29	38
LH–6	Relative amount RC (%)	—	8.31	—	4.29	0.50	1.61	0.73	84.56	100
	Kind No.	—	4	—	3	1	1	2	27	38
JM–92	Relative amount RC (%)	—	9.36	—	4.23	—	1.13	0.87	84.41	100
	Kind No.	—	4	—	3	—	1	2	24	34
C–6359	Relative amount RC (%))	4.41	7.33	—	1.20	—	10.82	0.55	75.70	100
	Kind No.	4	3	—	2	—	2	2	29	42
CH–1	Relative amount RC (%)	5.75	12.04	—	0.70	—	8.04	0.57	72.89	100
	Kind No.	5	4	—	1	—	2	2	28	42
CH–58	Relative amount RC (%)	3.47	11.76	—	1.60	—	9.85	0.48	72.84	100
	Kind No.	4	6	—	2	—	3	2	30	47
LX–66	Relative amount RC (%)	3.66	20.16	—	4.72	—	3.01	0.73	67.72	100
	Kind No.	3	6	—	3	—	3	2	23	40
JM–47	Relative amount RC (%)	10.99	26.17	—	2.05	2.36	6.38	0.75	51.30	100
	Kind No.	6	7	—	3	4	7	2	21	50
YH–115	Relative amount RC (%)	17.88	46.65	0.16	1.92	2.85	5.45	0.40	24.70	100
	Kind No.	7	9	1	3	5	6	2	16	49

Indicates that the flavoring substance has no found.

## 4 Discussion

This study evaluated differences in yield, nitrogen (N) accumulation and utilization, and processing-related quality traits among ten dryland winter wheat varieties widely cultivated on the Loess Plateau. The results reveal clear trade-offs and synergisms among yield components, N dynamics and quality attributes that have direct implications for variety selection and management in rainfed systems (Ren et al., 2019; Mbuthia et al., 2015). Clustering based on grain yield and protein content divided the varieties into three groups: high-yield medium-protein, medium-yield high-protein, and medium-yield medium-protein. High-yield medium-protein genotypes (e.g., YH–805, Yunhai–20410, Liangxing–66, Changhang–1) achieved greater grain yield primarily through increases in spike number and 1000-grain weight (Mbuthia et al., 2015). This confirms that, under dryland conditions, both sink capacity (spike number per unit area) and grain-filling ability after anthesis (which affects 1000-grain weight) are key drivers of yield. The particularly strong performance of YH–805—highest yield and highest grains per spike—indicates this cultivar combines effective tillering/ear formation with good post-anthesis assimilate partitioning (Noor et al., 2024, 2025).

However, the literature and our data indicate that the total number of grains per unit area often shows higher stability and heritability than 1000-grain weight. Thus, breeding or management that secures spike production (through early season vigor, optimized sowing density and soil moisture conservation) tends to deliver more stable yield gains in dryland environments than relying solely on grain-filling enhancements, which are more weather-dependent (Tao et al., 2019; Rasmussen and Thorup-Kristensen, 2016).

### 4.1 Nitrogen uptake, partitioning and protein implications

Varietal differences in N uptake efficiency, N harvest index (NHI) and post-anthesis N accumulation were pronounced and linked closely to final grain protein concentration (Cao et al., 2017). Varieties such as YH–618 and YH–805 exhibited higher NHI and greater N remobilization to grain after anthesis, explaining their elevated grain protein. These patterns underscore two complementary routes to high grain protein: (1) greater pre-anthesis N accumulation and conservative vegetative retention followed by remobilization, and (2) sustained post-anthesis N uptake from soil. For dryland wheat, where soil N availability and water-driven mineralization are constrained and variable, genotypes that efficiently capture and remobilize N under limited water are particularly valuable (Donmez and Yildirim, 2021; Miralles and Le Gouis, 2007). Practically, the clear genotype × year effects on N absorption efficiency and N utilization efficiency imply that varietal ranking for N-related traits may shift with seasonal conditions. Therefore, variety recommendations should be paired with location-season data and flexible management (e.g., timing of N applications) to realize both yield and protein targets (Wang et al., 2007; Tian et al., 2012).

### 4.2 Yield–quality trade-offs and processing quality

The inverse relationship between grain yield and protein concentration is a long-standing phenomenon and appeared in our grouping: high-yield genotypes tended toward medium protein, while some medium-yield genotypes had higher protein. This trade-off reflects dilution of grain protein under larger carbohydrate accumulation but can be managed partially through agronomy (timed N dressings, moisture conservation) and by selecting cultivars with favorable N partitioning (Luo et al., 2020; Yu et al., 2005). Processing quality is not determined by crude protein alone. Flour functionality (gluten quality), starch properties (gelatinization temperature, swelling power) and rheological behavior jointly define suitability for noodles, steamed bread or other products (Hu et al., 2014; Han et al., 2014). Our findings that starch swelling power and gelatinization characteristics correlate with noodle sensory scores highlight the need to evaluate both protein and starch traits in variety selection for end-use quality (Chen et al., 2015). Varieties such as YH–115 that are enriched in volatile aromatic compounds may offer added value in consumer preference but may require specific post-harvest or processing practices to retain aroma. Variety choice must balance yield potential with desired processing quality and local market demands. For bulk-food security goals, high-yield medium-protein genotypes like YH–805 and Yunhai–20410 are attractive; for premium end-products (e.g., high-protein flour for bread), medium-yield high-protein genotypes (e.g., YH–618) may be preferable. Management should be genotype-targeted (Noor et al., 2024). For high-yielding varieties, practices that protect early-season tiller survival and conserve fallow-season water (mulch, stubble retention, optimized sowing date and density) will help maximize spike number and grain number per unit area. For high-protein targets, split N applications with an emphasis on late-season N (if moisture permits) can increase grain protein without overly penalizing yield. Screening for both N-use traits and processing-relevant starch and gluten properties should be part of breeding and on-farm evaluation pipelines. Given the year × cultivar interactions observed for N traits, multi-year, multi-site testing in representative dryland conditions remains essential.

## 5 Conclusions

The present study establishes that the fundamental trade-off between wheat yield and protein content stems from varietal differences in variety processes, including spike formation, grain filling, and nitrogen uptake/remobilization. Our findings clearly prove that varietal action is dictated by diverging strategies in resource allocation and temporal arrangement, while breeders can select for varieties such as YH–805 that optimize resource translocation for maximum yield, or YH–618 for protein content; variety YH–115 represents a valuable genetic resource for breeding programs aimed at enhancing premium quality traits without compromising moderately high yield and protein levels. Varietal differences in spike formation, grain-filling capacity, and N uptake/remobilization explain much of the observed variation in yield and protein in dryland wheat genotypes. Targeted selection and management—matching cultivar traits (e.g., high spike fertility vs. strong N remobilization) to production objectives (yield stability vs. high protein/processing quality) and to local water–N dynamics—will be the most effective route to raising both productivity and grain quality on the Loess Plateau’s drylands. Finally, the choice of variety depends on the specific agricultural and market objectives, whether quantity or culinary quality. Future research should validate these adaptive N thresholds across diverse soil types and crop rotation systems.

## Data Availability

The original contributions presented in the study are included in the article/**Supplementary Material**, further inquiries can be directed to the corresponding author/s.
