# Supplementary material for: Cultivation practices and soil water storage effects on grain yield and quality of winter wheat in the Loess Plateau, China
Source: Front Plant Sci. 2026 Jan 23;16:1735429. doi: 10.3389/fpls.2025.1735429 (PMC12875960; doi:10.3389/fpls.2025.1735429)
Supplement: Supplementary file 1 [file Table1.docx]

| Table 2S. Information of different dryland wheat cultivars | | | | |
| --- | --- | --- | --- | --- |
| **No** | **Name** | **Variety source** | **Wheat quality type** | **Breeding institutions** |
| 1 | YH–20410 | JM54/C5613 | Strong gluten | Cotton Research Institute,SXAU |
| 2 | YH–618 | YH92-18/XC9 | Strong gluten | Cotton Research Institute,SXAU |
| 3 | JM–92 | 3333LY6148/JM33 | Strong gluten | Wheat Research Institute,SXAU |
| 4 | LH–6 | YM49/SN25 | Medium-gluten | [Academy of Agricultural Sciences of LY](https://baike.baidu.com/item/%E6%B4%9B%E9%98%B3%E5%B8%82%E5%86%9C%E4%B8%9A%E7%A7%91%E5%AD%A6%E7%A0%94%E7%A9%B6%E9%99%A2/6321479) |
| 5 | C–6359 | 94-5383/82230-6 | Medium-gluten | Millet Research Institute,SXAU |
| 6 | CH–1 | CW131/SY225 | Medium-gluten | Changwu County agricultural technology Center |
| 7 | CH–58 | 112/PH82-2CW112/PH82-2C | Medium-gluten | Agricultural Technology Extension Center of Changwu County of SXAU |
| 8 | LX–66 | J91102/J935031 | Medium-gluten | Liangxing seeds company of SD |
| 9 | JM–47 | H522/K37-20/12057 | Strong gluten | Cotton Research Institute,SXAU |
| 10 | YH–115 | Y361/L139 | Strong gluten | Cotton Research Institute,SXAU |
